# Percutaneous pericardiocentesis using the apical approach: case series and review of the literature

**DOI:** 10.1186/s43044-024-00537-8

**Published:** 2024-08-16

**Authors:** Malek Alaiwah, Ahmed Hassanin, Danish Abbasi, Hamza Rayes, Zahra Hassan, Mu’nes Albadaineh, Munthir Mansour, Subodh Devabhaktuni, Tushar Tarun, Srikanth Vallurupalli, John P. Mounsey, Subhi J. Al’Aref

**Affiliations:** 1 Department of Cardiology, University of Arkansas for Medical Sciences, 4301 W, Markham St, Little Rock, AR 72205 USA; 2 College of Medicine, University of Arkansas for Medical Sciences, Little Rock, AR USA

**Keywords:** Pericardial effusion, Cardiac tamponade, Pericardiocentesis, Apical approach, Echocardiography

## Abstract

**Background:**

Pericardiocentesis offers a definitive diagnostic and a life-saving therapeutic modality through removal of pericardial fluid and relief of high intrapericardial pressure. Percutaneous pericardiocentesis has been performed via different approaches depending on different institutional experiences. In this paper, we present our institutional experience and review the current literature of the different approaches for performing percutaneous pericardiocentesis.

**Materials and methods:**

We evaluated consecutive patients who underwent echocardiographic-guided pericardiocentesis via the apical approach for pericardial effusion between the period of April 1st, 2022, and April 1st, 2023, at University of Arkansas for Medical Sciences (UAMS). Health records were reviewed for clinical presentations, available imaging findings, procedural outcomes, and short-term follow up.

**Results:**

A total of eight consecutive cases of pericardiocentesis via the apical approach were found. Seven were successful. No complications were reported. Six patients had evidence of tamponade physiology on echocardiogram.

**Conclusion:**

Historically, pericardiocentesis has been most performed via the subxiphoid approach. However, an ultrasound-guided apical approach offers a safe and effective alternative and may be preferable in patients with challenging anatomies.

**Supplementary Information:**

The online version contains supplementary material available at 10.1186/s43044-024-00537-8.

## Background

The occurrence of a pericardial effusion has a wide variation in clinical presentation, ranging from an incidental finding on imaging to hemodynamic collapse. Pericardiocentesis offers a definitive diagnostic and therapeutic modality through removal of the pericardial fluid and relief of elevated intrapericardial pressure [1].

Many techniques have evolved to improve percutaneous pericardiocentesis safety and outcomes since it was first described by Frank Schuh in 1840 [2–4]. In the early 1980s, two-dimensional echocardiography-assisted pericardiocentesis was first described [5]. Numerous studies have demonstrated the efficacy and safety of echocardiography-guided pericardiocentesis [6, 7]. Traditionally, percutaneous pericardiocentesis is performed via a subxiphoid approach as it has been considered the safest approach in the absence of imaging guidance [8, 9]. With the increasing use of echocardiography, the practice patterns have evolved into relying mainly on echocardiographic findings in choosing the procedural approach, although many institutions still use the subxiphoid approach as their default approach [7–9, 11, 12]. In our institution we choose pericardiocentesis approach mainly based on echocardiographic and other imaging findings, where the apical approach is commonly the optimal site to use. In this article, we present our institutional experience with echocardiography and fluoroscopy-guided pericardiocentesis via the apical approach, along with a literature review on the safety, efficacy, and potential advantages of this approach.

## Main text

### Material and methods

We evaluated consecutive patients who underwent echocardiographic-guided pericardiocentesis via the apical approach for pericardial effusion between the period of April 1st, 2022, and April 1st, 2023, at University of Arkansas for Medical Sciences (UAMS). No patients were excluded. Electronic health records were reviewed for clinical profiles, available imaging, echocardiography findings, procedural details, outcomes, and short-term follow-up. The procedure was determined to be successful if there was no or trivial pericardial effusion noted on repeat transthoracic echocardiography (TTE) done within 24 h of the index procedure. A complication was defined as the occurrence of one of the following events: myocardial puncture requiring emergent surgery, liver injury, hematoma, pneumothorax, tension pneumopericardium, arrhythmia, or peri-procedural death.

### Procedural details

1. Echocardiography images were obtained prior to the procedure. Other available imaging modalities were reviewed to evaluate for the optimal percutaneous approach, based on distance between skin entry and largest fluid pocket as along with intervening structures.
2. The optimal entry site was identified usually 1–2 cm lateral to the apex beat within the 5–7th intercostal spaces. Then, the area was anesthetized with lidocaine.
3. Under continuous ultrasound (US) guidance, a micro-puncture needle was advanced with negative pressure superior to the upper border of the corresponding rib, to avoid injury to the intercostal neurovascular bundle. As the needle enters the pericardium, a “giveaway” sensation is typically felt. After needle position was confirmed in the pericardial sac both with echocardiography and fluoroscopy, the micro-puncture wire was advanced followed by the 4-Fr micro-puncture sheath. Intra-pericardial position was confirmed using agitated saline seen on bedside echocardiography.
4. A J-tip 0.035″ × 145 cm Amplatz Super Stiff (Boston Scientific, Marlborough, MA) wire was advanced into the pericardium under fluoroscopic guidance and a dilator was used to dilate the track.
5. An 8-Fr High Flow Straight Catheter (Boston Scientific, Marlborough, MA) was then advanced, and drain was connected to a collecting bag and was sutured in place.
6. All effusions were attempted to be drained completely. Post-procedure echocardiography was done to assess for the presence of a residual pericardial effusion.
7. The drain was left in situ in almost all therapeutic cases and was later removed once drain output was less than 25 cc over 24 h, with no recurrence of pericardial effusion noted on echocardiography.

### Results

Patient and procedural characteristics are described in Table 1, and details of two of cases of the series are described in Tables 2 and 3. In total, there were eight consecutive cases of pericardiocentesis via the apical approach. The median age for patients was 54 years old (interquartile range 38–71). Four patients were male, and four were female. Six cases had echocardiographic evidence of tamponade physiology, and the other two cases were done for symptomatic large pericardial effusions. Four patients were fully anticoagulated on presentation for various indications (three patients for pulmonary embolus (PE) and one for history of deep vein thrombosis). Seven patients had circumferential pericardial effusions. The pericardial drain was left in place in six cases. The longest duration of pericardial drain retention was 144 h, which occurred in a patient with end stage renal disease (ESRD). The aforementioned patient was considered for pericardial window, but eventually was transitioned to comfort care. There were no immediate or late complications in any of the patients. The procedure was successful in seven cases. In one case with a large loculated pericardial effusion, multiple attempts to drain the effusion only resulted in 120 ml output. Subxiphoid approach was not attempted due to small fluid pocket. Due to multiple loculations and inability to drain most of the pericardial effusion, the procedure was aborted, and the patient underwent a pericardial window the following day.

**Table 1:** Patient and procedural characteristics

Patient and procedural characteristics:	
Number of patients	8
Age; median (interquartile range in years)	53.5 (38–71)
Male; *n* (%)	4 (50%)
Pericardial effusion location	
Circumferential effusion; *n* (%)	7 (88%)
Loculated effusion; *n* (%)	1 (12%)
Pericardial effusion characteristics	
Serous; *n* (%)	3 (38%)
Sanguineous; *n* (%)	3 (38%)
Serosanguinous; *n* (%)	2 (25%)
Etiologies:	
Uremia; *n* (%)	4 (50%)
Malignancy; *n* (%)	2 (25%)
Viral; *n* (%)	1 (12%)
Idiopathic or indeterminate; *n* (%)	1 (12%)
Therapeutic anticoagulation; *n* (%)	4 (50%)
Amount of drained pericardial fluid; median (interquartile range)	955 (660–1755) ml
Drain placement duration; median (interquartile range)	32 (13–46.5) hours
Success rate %; (*n*)	88% (7)
Complication rate; (*n*)	0% (0)

*n*  number, *IQR*  interquartile range

**Table 2:** Case number 1

Case #1	
Brief history and physical examination	A 29-year-old female with neurofibromatosis, who presented with worsening shortness of breath, and hypoxia requiring supplemental oxygen. She underwent a computed tomography (CT) scan of the chest and was found to have a pulmonary embolus, along with a large pericardial effusion and a large mass along the right heart border. The patient was started on anticoagulation for PE. TTE confirmed large circumferential pericardial effusion with evidence of tamponade physiology
Indication	Therapeutic and diagnostic
Pericardial effusion size and location on transthoracic echo	Large circumferential
Anticoagulation use	Therapeutic heparin
Type of pericardial effusion	Sanguineous. Figure 1
Amount drained	700 ml
Etiology	Malignant
Duration of drain placement	38 h
Complications	None
Outcomes	No reaccumulating of the pericardial effusion was noted on repeat TTE, 2 days after the index procedure the pericardial drain was removed
Comments	This patient had a metastatic sarcoma with a large mass along the right heart border that was engulfed within the large pericardial effusion, making subxiphoid approach for pericardiocentesis risky, especially while on therapeutic anticoagulation. In this case using available CT chest and echocardiography images was useful, which helped in making the determination that the apical approach was the safest site for skin entry. Figures 1, 2 and 4

**Fig. 1:**
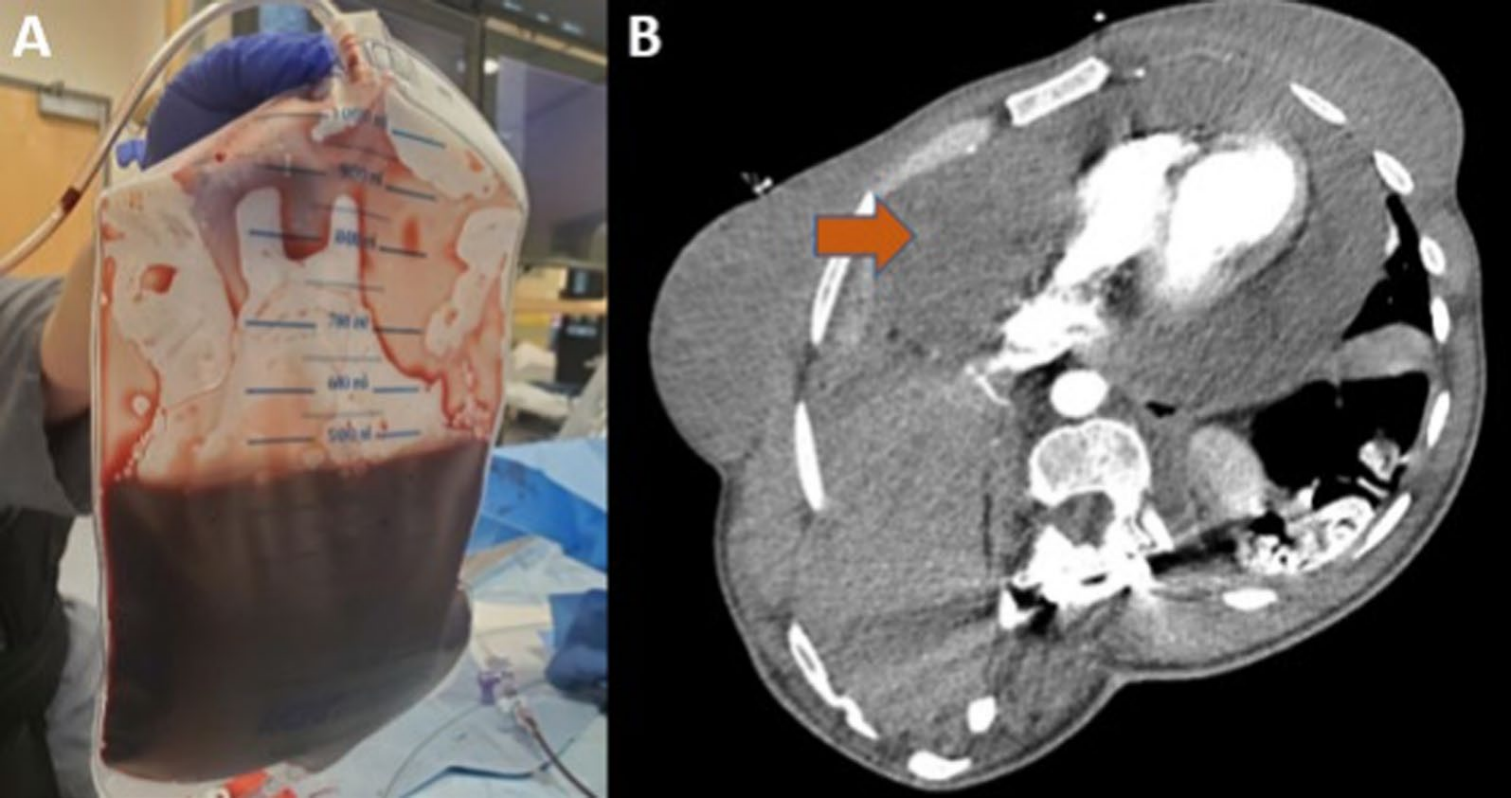
Case #1; **A**: Serosanguinous pericardial fluid **B**: Axial section from CT scan of the chest showing a large mass (orange arrow) in the anterior mediastinum along the right heart border

**Fig. 2:**
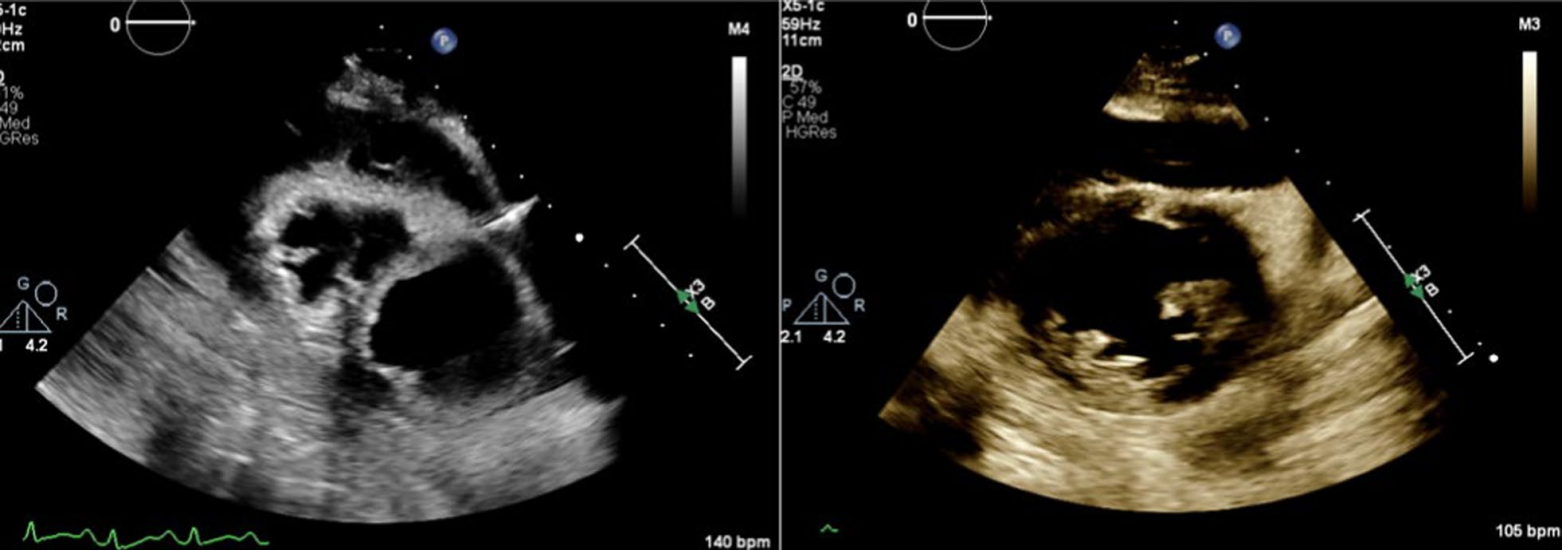
Case #1; TTE parasternal short-axis view before (left image) and after (right image) pericardiocentesis

**Table 3:** Case number 2

Case #2	
Brief history and physical examination	A 78-year-old female with hypertension presented with shortness of breath. Patient was hemodynamically stable but hypoxic requiring supplemental oxygen. The patient had received one dose of therapeutic enoxaparin the morning of the procedure for presumed PE. TTE showed large circumferential pericardial effusion with echocardiographic evidence of tamponade physiology
Indication	Therapeutic and diagnostic
Pericardial Effusion size and location on transthoracic echo	Large circumferential
Anticoagulation use	Therapeutic enoxaparin
Type of pericardial effusion	Serosanguinous
Amount drained	620 ml
Etiology	Viral
Duration of drain placement	26 h
Complications	None
Outcomes	Trivial pericardial effusion was noted on repeat TTE, 2 days after pericardial drain was removed
Comments	On TTE, a substantial portion of the liver was noted to be between the skin and the pericardium, which made using the subcostal approach risky in terms of liver injury and bleeding complications (Fig. 3). The apical view showed a large pocket of pericardial effusion without overlying lung tissue (Fig. 4)

**Fig. 3:**
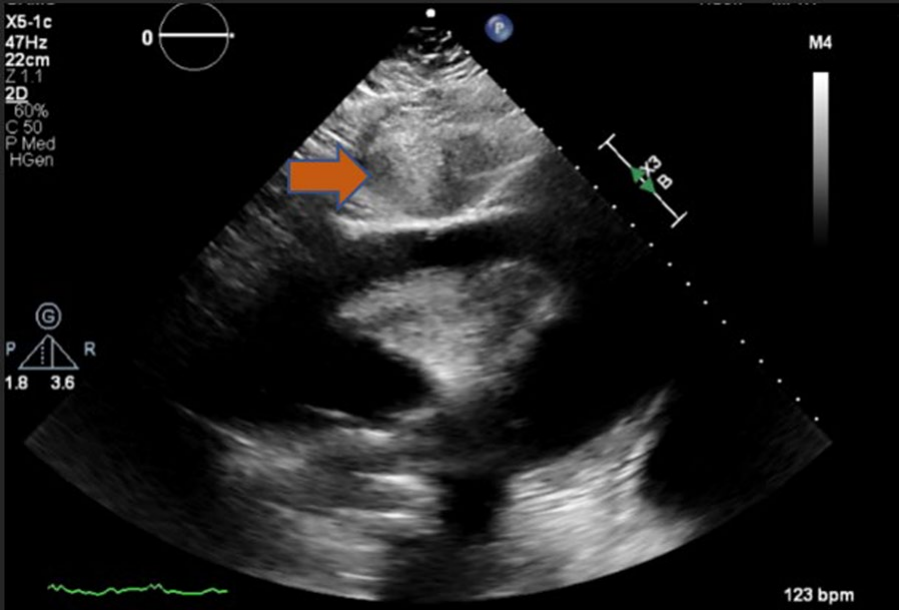
Case 2; TTE showing a subxiphoid view; a substantial portion of the liver (orange arrow) noted between the skin and the pericardial sac

**Fig. 4:**
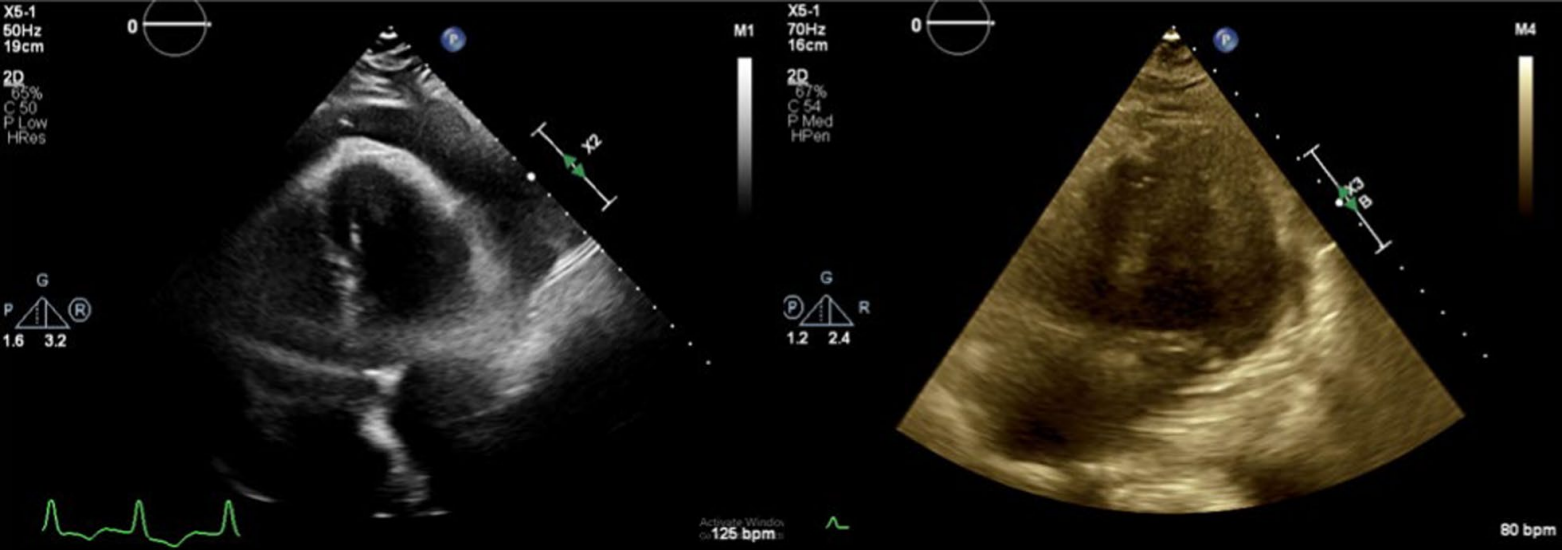
Case 2; TTE apical four-chamber view before (left image) and after (right image) pericardiocentesis

### Discussion

Percutaneous pericardiocentesis is associated with significant risks, especially when performed in emergency situations [4]. The rates of complications cited in the literature range from 1.2 to 1.6% and include myocardial puncture, pneumothorax, tension pneumopericardium, and arrhythmia [3, 6, 13, 14]. Using imaging guidance improved significantly procedural outcomes. Echocardiography is the most used modality in assisting pericardiocentesis, in part due to its wide availability, ease of use, and association with good outcomes as demonstrated by multiple investigations [3–5, 7].

**Fig. 5:**
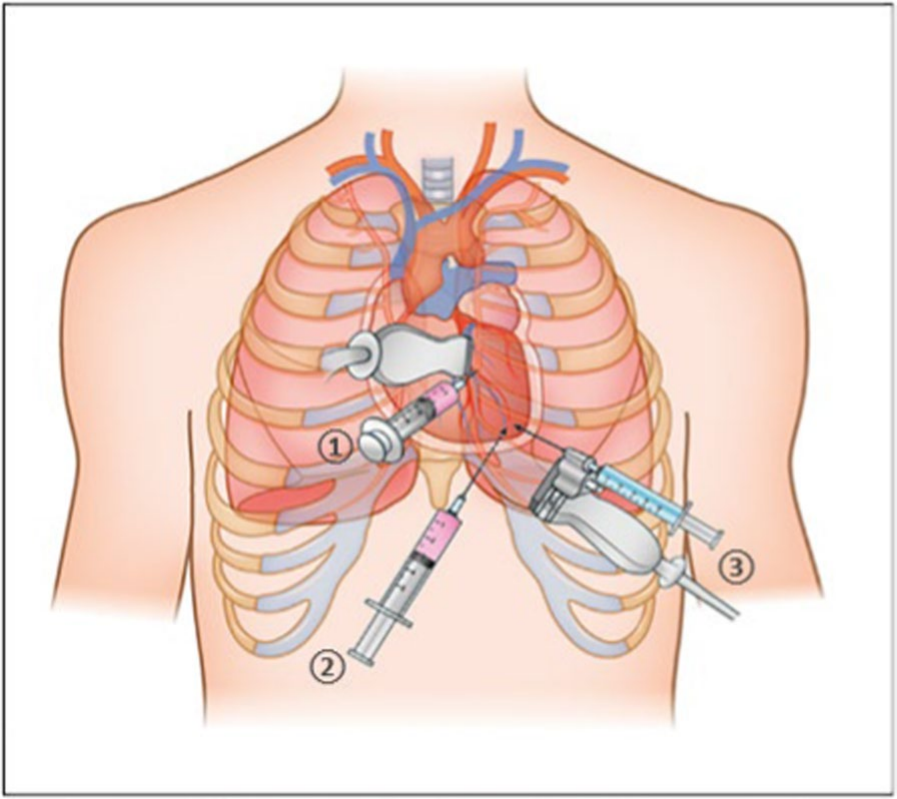
Percutaneous pericardiocentesis approaches; ① parasternal approach, ② subxiphoid approach and ③ apical approach [10]

Three main puncture sites can be used in echocardiography-guided pericardiocentesis: apical or para-apical, subxiphoid, and parasternal approach (Fig. 5) [10]. The subxiphoid approach has been the standard route used before the emergence of echocardiography and remains the most used site in many institutions because it has historically been considered the safest approach [7–9, 11]. The ideal approach should be selected based on the largest pericardial effusion pocket along with the shortest distance between the skin and pericardial space with no vital structures in between [6]. There are two methods of utilizing echocardiography in pericardiocentesis: the echocardiography-assisted method, where the operator memorizes the needle entry site and trajectory without continuous echocardiography monitoring. The other approach is the echocardiography-guided method, where the operator uses echocardiography for continuous monitoring to ensure needle entry to the pericardial space [4].

Using an apical approach offers several potential advantages. Usually, the apical approach is the shortest path between skin and pericardial space, with no or very minimal lung tissue laying in between. And with echocardiography guidance, the operator can confidently choose the optimal entry site, as ultrasound waves will not pass through air easily as is reflected on the images obtained [6, 10]. Additionally, it is not uncommon to encounter an enlarged liver or a tumor obscuring the subxiphoid window as we highlight in the two cases described in Tables 2 and 3. The first patient had metastatic sarcoma with a large mass attached to the right ventricle (RV), and the second patient had hepatomegaly. In both cases, it would be challenging to maneuver the needle away from these structures safely. These considerations become important when patients are anticoagulated, or have certain coagulopathies, as observed in four patients in our series. Also, given that left ventricle (LV) is thicker than the right ventricular (RV) wall, LV injury is more likely to self-seal after accidental puncture [4, 6, 7, 10].

Nevertheless, using the apical approach can be impractical in certain situations. First, loculated pericardial effusions away from the apex may not be accessible via the apical approach, as was the case in one of our patients (case #4 in index A). Also, the absence of good apical windows due to body habitus, musculoskeletal deformities, or previous thoracic surgeries would preclude using the apical entry site. A comparison of the advantages and disadvantages of each approach is listed in Table 4.

**Table 4:** Percutaneous pericardiocentesis approaches, advantages and disadvantages

Approach	Description	Advantages	Disadvantages/Challenges
Apical or para-apical	Needle insertion site is within the 5th, 6th or 7th intercostal space, usually lateral to the apex by 1–2 cm	Usually is the shortest pathway between skin and pericardial space Minimal intervening structures between skin and pericardial space LV wall is thicker compared to RV which makes it more likely to seal after an accidental puncture	Poor apical echocardiography windows in some individuals higher risk of LV injury Theoretically a higher risk of pneumothorax (although no numbers are published in the literature)
Subxiphoid	Needle insertion site is between xiphisternum and left costal margin	Away from lung tissue (lower risk of pneumothorax)	Longer path to reach the pericardial space High risk of liver injury or entering the peritoneal cavity Potential risk of right atrial injury
Parasternal	Needle insertion site is in the left 5th intercostal space, next to the sternal margin	Can use regular cardiac or high frequency linear probe to guide the procedure	Risk of pneumothorax and damage to the internal thoracic vasculature

Several large case series of echocardiography-guided pericardiocentesis have been published, which provide an insight into the variable institution-specific considerations and outcomes as it relates to the apical approach for percutaneous pericardiocentesis, [6] versus the subxiphoid approach [7, 12]. In a large echocardiography-guided pericardiocentesis series, published in 2002, Tsang et al. [6] presented 1127 cases they encountered over 21 years. Almost 65% of these cases were done using the apical approach versus 19% using subxiphoid. The success rate reached 97% in this series and the rate of major complications was 1.2% (1 death due to RV puncture, 5 patients had non-fatal myocardial injury requiring surgery, 5 had pneumothorax, and 1 had intercostal vessel injury requiring surgery). In another series published in 2014 by Akyuz et al. [7] that included 301 patients over a 10-year span, a subxiphoid approach was used in 85% of cases and an apical approach in 15%. The overall success rate was 97%, and the major complications rate was 1.3% (3 patients had myocardial injury and 1 had pneumothorax). Finally, in a series by Haddad et al. [12] published in 2015 that included 212 patients with cancer requiring percutaneous pericardiocentesis, a subxiphoid approach was used in 63% of cases and an apical approach in 37%. The overall success rate was 99%, and the major complications rate was 2% (1 patient had liver laceration requiring surgical repair, 1 had intercostal artery laceration requiring surgery, and 1 had pneumothorax). In none of these studies was there a breakdown of success or complications rates of one approach versus the other. Upon reviewing percutaneous pericardiocentesis procedures that were done via the subxiphoid approach at UAMS, between the period between April 1st, 2022, until April 1st, 2023, we found total of 7 patients. Success and complications rate were comparable to the apical approach pericardiocentesis that were done at UAMS during the same period. The high success rates in the aforementioned series, as well as ours, and the absence of guideline statements [1] on an “optimal” approach for percutaneous pericardiocentesis further emphasize the importance of individualizing the approach for each patient.

## Conclusion

Pericardiocentesis via the apical approach has its potential benefits and limitations. Each case should be evaluated individually based on their clinical and anatomical characteristics, while also accounting for the operator’s and institutional experience. Using available images modalities, including echocardiography, computed tomography and fluoroscopy, can aid in achieving optimal success and safety outcomes within the realm of percutaneous pericardiocentesis.

### Supplementary Information

Supplementary Material 1.

## Data Availability

Not applicable.

## Abbreviations

UAMS: University of Arkansas for Medical Sciences
TTE: Transthoracic echocardiography
US: Ultrasound
IQR: Interquartile range
*n*: Number
RV: Right ventricle
LV: Left ventricle
CT: Computed tomography
