# Supplementary material for: Percutaneous pericardiocentesis using the apical approach: case series and review of the literature
Source: Egypt Heart J. 2024 Aug 16;76:106. doi: 10.1186/s43044-024-00537-8 (PMC11329492; doi:10.1186/s43044-024-00537-8)
Supplement: Supplementary file 1 — Supplementary Material 1. [file 43044_2024_537_MOESM1_ESM.docx]

Index A

| Case #3 | |
| --- | --- |
| Brief history and physical exam | A 38-year-old male with hypertension and morbid obesity who presented with worsening shortness of breath over few days. He presented with low systolic blood pressure (98/78 mm Hg), tachycardia to 111 bpm, tachypnea, with normal pulse oximetry levels. Electrocardiogram (ECG) showed diffuse ST segment elevation and ST depression in aVR lead, suggestive of acute pericarditis. TTE was done which demonstrated large circumferential pericardial effusion with echocardiographic evidence of pericardial tamponade. |
| Indication | Therapeutic and diagnostic |
| Pericardial Effusion size and location on transthoracic echo | Large circumferential |
| Anticoagulation use | None |
| Type of pericardial effusion | Serous |
| Amount drained | 710 ml |
| Etiology | Idiopathic |
| Duration of drain placement | 26 hours |
| Complications | None |
| Outcomes | No reaccumulating pericardial effusion was noted on repeat TTE, 1 day after index procedure the pericardial drain was removed. |
| Case #4 | |
| Brief history and physical exam | A 67-year-old female with hypertension, chronic kidney disease, and congenital intellectual disability who presented to the emergency department after an unwitnessed fall and knee pain. She presented with elevated blood pressure, normal heart rate, and normal pulse oximetry levels. Chest Xray was done for chest pain and showed an enlarged cardiac silhouette. Bedside echocardiography showed incidental large pericardial effusion. Formal TTE confirmed the presence of loculated large pericardial effusion with echocardiographic evidence of pericardial tamponade. |
| Indication | Therapeutic |
| Pericardial Effusion size and location on transthoracic echo | Large loculated |
| Anticoagulation use | None |
| Type of pericardial effusion | Serous |
| Amount drained | 120 ml |
| Etiology | Uremic |
| Duration of drain placement | 0 hours |
| Complications | None |
| Outcomes | Post pericardiocentesis TTE showed moderate to large loculated pericardial effusion without evidence of tamponade physiology. Multiple unsuccessful attempts were made to drain the residual pericardial fluid. The patient underwent pericardial window surgery the following day. |
| Case #5 | |
| Brief history and physical exam | A 42-year-old male with hypertension, end-stage renal disease on hemodialysis and deep vein thrombosis on apixaban who presented to the emergency department with worsening shortness of breath. He presented with elevated blood pressure, tachycardia to 120 bpm, with pulse oximetry reading of 89% on room air. TTE showed large pericardial effusion with tamponade physiology. CT chest was negative for pulmonary embolus. |
| Indication | Therapeutic |
| Pericardial Effusion size and location on transthoracic echo | Large circumferential |
| Anticoagulation use | Apixaban |
| Type of pericardial effusion | Sanguineous |
| Amount drained | 1900 ml |
| Etiology | Uremic |
| Duration of drain placement | 43 hours |
| Complications | None |
| Outcomes | Trivial pericardial effusion was noted, 1 day after the index procedure the drain was removed. TTE repeat 3 months after index procedure showed only trivial pericardial effusion. |
| Case #6 | |
| Brief history and physical exam | A 38-year-old male with hypertension, end-stage renal disease on hemodialysis presented with progressive shortness of breath. He was found to have a large pericardial effusion without tamponade physiology on TTE. |
| Indication | Therapeutic |
| Pericardial Effusion size and location on transthoracic echo | Large circumferential, but predominantly posteriorly located |
| Anticoagulation use | None |
| Type of pericardial effusion | Serous |
| Amount drained | 1200 ml |
| Etiology | Uremic |
| Duration of drain placement | 0 hours |
| Complications | None |
| Outcomes | Post procedure TTE showed no pericardial effusion. |
| Case #7 | |
| Brief history and physical exam | A 75-year-old male with hypertension, end-stage renal disease on hemodialysis, status post implantable cardioverter defibrillator (ICD) placement for secondary prevention, known to have moderate pericardial effusion who was admitted for altered mental status and developed ventricular tachycardia cardiac arrest. Patient was persistently hypotensive after return of spontaneous circulation. Repeat TTE after cardiac arrest showed significant increase in pericardial effusion, but without tamponade physiology. |
| Indication | Therapeutic |
| Pericardial Effusion size and location on transthoracic echo | Large circumferential, but predominantly posteriorly located |
| Anticoagulation use | None |
| Type of pericardial effusion | Sanguineous |
| Amount drained | 2600 ml |
| Etiology | Uremic |
| Duration of drain placement | 144 hours |
| Complications | None |
| Outcomes | Post procedure TTE showed trivial pericardial effusion. Drain continued to put significant output on daily basis. Pericardial window was considered, but given patient overall clinical picture, he was transitioned to comfort care and died. |
| Case #8 | |
| Brief history and physical exam | A 65-year-old female with adenocarcinoma of the lung, pulmonary embolus (on Rivaroxaban), who presented for shortness of breath. She was normotensive, tachycardic to 105 bpm, tachypneic requiring supplemental oxygen. She was found to have large pericardial effusion on bedside TTE with tamponade physiology. |
| Indication | Therapeutic |
| Pericardial Effusion size and location on transthoracic echo | Large circumferential |
| Anticoagulation use | Rivaroxaban |
| Type of pericardial effusion | Sanguineous |
| Amount drained | 1610 ml |
| Etiology | Malignant |
| Duration of drain placement | 50 hours |
| Complications | None |
| Outcomes | Post procedure TTE showed trivial pericardial effusion. Drain continued to have significant daily output. Pericardial window was done 3 days after index procedure. |
